# Non-RBD peptides of SARS-CoV-2 spike protein exhibit immunodominance as they elicit both innate and adaptive immune responses

**DOI:** 10.1016/j.heliyon.2024.e39941

**Published:** 2024-10-29

**Authors:** Deepika Rathore, Preeti Chauhan, Anvesh Bonagiri, Lekha Gandhi, Deepti Maisnam, Ramesh Kumar, Anupama T. Row, M.M. Kesavulu, Musturi Venkataramana

**Affiliations:** a Department of Biotechnology and Bioinformatics, School of Life Sciences, University of Hyderabad, Gachibowli, 500046, Hyderabad, Telangana State, India; b Health Centre, University of Hyderabad, Gachibowli, 500046, Hyderabad, Telangana State, India; c Department of Basic Sciences and Humanities, Sree Vidyanikethan Engineering College, Tirupati, Andhra Pradesh, India

**Keywords:** SARS-CoV-2, COVID-19, Vaccines, Spike protein, Receptor binding domain, Peptide vaccines

## Abstract

Severe acute respiratory coronavirus-2 (SARS-CoV-2) emerged in 2019 as a new virus and caused worldwide outbreaks, quickly turning into a pandemic disease called coronavirus disease-19 (COVID-19). All the existing methodologies were used for developing vaccines for this virus. But sporadic infections of this virus and the emergence of new strains to date suggest the incomplete protection offered by the developed vaccines and the need for new research. In this direction, we identified five epitopes present in the non-RBD region and on the surface of the spike protein by in silico analysis. They are epitope I (aa 80–90), epitope II (aa 262–270), and a small protein with three epitopes (aa 1059–1124). Antigenicity scores of these epitopes were found to be higher than the full length spike protein and its RBD region. These epitopes showed high conserveness across the emerging strains, high immunogenicity, non-toxicity, no homology with human sequences and high affinity for MHC class I & II molecules. Antibodies raised against these epitopes interacted with the bacterially expressed spike protein in western blotting. The antiserum of COVID-19 recovered participants reacted with the developed epitopes (small protein). Furthermore, in the presence of the respective antiserum and COVID-19 convalescent serum, these epitopes successfully fixed the complement, implying a possible role in innate immunity. The epitopes were also found to activate the peripheral blood mononuclear cells (PBMCs) isolated from the blood samples of COVID-19 recovered/vaccinated participants, suggesting a possible role in adaptive immunity. The need for the new SARS-CoV-2 vaccines is further highlighted in light of current reports about the side effects of a developed vaccine (AstraZeneca) and the circulating new strains. The epitopes presented in this study represent the potential immunogens and expect certain pitfalls of the existing vaccines would be sealed.

## 1 Introduction

Severe acute respiratory syndrome coronavirus-2 (SARS-CoV-2) has been causing the devastating disease known as coronavirus disease-19 (COVID-19) for the past four years. This virus was reported in the year 2019 in the city of Wuhan, China and reached a world pandemic at the fastest rate in the history of human virus outbreaks. The infections were spread to 230 countries, infecting more than 774 million people, with a death toll of 7 million as of April 2024 [1]. Before SARS-CoV-2 was identified as the seventh human coronavirus, there were six human coronaviruses (HCoVs) reported: HCoV-OC43, HCoV-229E, HCoV-HKU1, HCoV-NL63, SARS-CoV-1 and Middle East Respiratory Syndrome coronavirus (MERS-CoV). Among the above HCoVs, SARS-CoV-1, MERS-CoV, and SARS-CoV-2 have emerged as novel zoonotic CoVs that are highly pathogenic and cause lethal human diseases. Despite the fact that SARS-CoV-1, MERS-CoV, and SARS-CoV-2 are members of the same virus family, i.e., the Coronaviridae, SARS-CoV-2 exhibits significant divergence [2]. SARS-CoV-2 was considered a distinct virus based on its percent identity with other beta coronaviruses in the open reading frame 1a/b, which is below 90 % identity with any of the reported HCoVs [3]. Notably, SARS-CoV-2 was closely related (87–92 % identity) to two bat-derived SARS-like coronaviruses (bat-SL-CoVZC45 and bat-SL-CoVZXC21) collected in 2018 in Zhoushan, Eastern China, but were more distant from SARS-CoV-1 (79 %) and MERS-CoV (50 %). COVID-19 has less severe pathogenesis than previously known human CoVs, but its higher transmission has resulted in a global threat, as evidenced by the continuously increasing number of confirmed cases [4,5]. All the SARS-CoVs exhibit different mechanisms to evade the host immune response and hence cause severe disease [6]. Unlike other respiratory diseases that have a quadratic (U-shaped) lethality curve (killing infants and the elderly but sparing adults), SARS-CoV-2 has a lethality that continuously rises with age (sparing children but mainly killing the elderly) [7]. Although the percent of mortality of SARS-CoV-2 (3–4%) is less than that of SARS-CoV-1 (15 %) and MERS-CoV (34 %), SARS-CoV-2 caused incomparable deaths in total. This is due to the specific point mutations attained in spike protein, which might promote efficient transmission and immune escape. In this direction, the SARS-CoV-2 variants, viz. Alpha (B.1.1.7), Beta (B.1.351), Gamma (P.1), and Delta (B.1.617.2), its variants (AY 1, 2, and 3), Omicron, and Deltacron, have emerged [7,8]. These variants show amino acid mutations mostly in the receptor binding domain (RBD) of the spike protein [9]. There was a peak in the number of cases across the globe immediately after the emergence of some of these new variants [10,11]. The genome of SARS-CoV-2 is a positive-sense single-stranded RNA of size ∼30 kb, the largest known viral RNA, and codes for a large polyprotein. Two-thirds of the viral genome encodes for two overlapping open reading frames (ORF1a and ORF1ab), which encode 16 non-structural proteins. The remaining one-third encodes for four structural proteins: spike (S), membrane (M), envelope (E), and nucleocapsid (N). The S protein is involved in the virus entry into the host cells; M protein interacts with N protein, which is embedded in the envelope. SARS-CoV-1 and MERS-CoV virus characteristics and pathogenicity studies were used as templates for understanding SARS-CoV-2 and to develop vaccines/drugs. Homology modelling methods show that similar to SARS-CoV-1, SARS-CoV-2 also binds to the receptor called the angiotensin-converting enzyme-2 (ACE2) domain with variable amino acid residues of S protein [4]. The S protein, spanning 1273 amino acids, comprises a signal peptide (aa 1–13), S1 subunit (aa 14–685), and S2 subunit (aa 686–1273). The S1 subunit includes an N-terminal domain (aa 14–305) and a receptor-binding domain (RBD; aa 319–541). S2 subunit features a fusion peptide (FP) (aa 788–806), heptapeptide repeat (HR1) (aa 912–984), HR2 (aa 1163–1213) sequences, a transmembrane (TM) domain (aa 1213–1237), and a cytoplasmic domain (aa 1237–1273).

COVID-19 disease manifests common symptoms like fever, diarrhoea, nausea, cough, dyspnoea, fatigue, myalgia, pneumonia, upper respiratory tract infections, and organ dysfunction [e.g., shock, acute respiratory distress syndrome (ARDS), acute cardiac injury, and acute kidney failure] [12]. The need for the vaccine was an emergency during the COVID-19 pandemic due to the rapid increase in active cases and deaths. The developed vaccine candidates include RNA, DNA, inactivated, protein subunits, replicating and non-replicating viral vectors, etc. [8,13,14]. However, certain pitfalls were not addressed in the developed vaccines concerning their safety and efficacy. The vaccines were developed using the original wild-type strain from the early phase of the pandemic, resulting in differences in the S protein sequence compared to emerging variants of concern (VoCs). This phenomenon led to the emergence of new strains and the increase in the number of cases worldwide, in spite of several rounds of vaccination. Because of its immunopathological properties, whole spike protein has been discouraged from being used as a vaccine candidate [[15], [16], [17]]**.** In macaques, the anti-S-protein antibodies caused the antibody dependent enhancement (ADE) in SARS-CoV-1 and similar concerns were expected in the case of SARS-CoV-2 as well [18,19]. ADE has been reported with the use of both SARS-CoV-2 vaccines and monoclonal antibody administrations [[20], [21], [22]]. Hence, it is necessary to identify and exclude the ADE inducible epitopes. Neutralization titers against certain variants of concern (VOCs) are reduced in individuals vaccinated with mRNA and adenoviral vector-based vaccines due to the use of full-length spike protein in its unstable conformation. Antibodies produced by certain mRNA vaccines, which rely on prefusion spike protein confirmation, also exhibit decreased effectiveness against VoCs. Moreover, immunity provided by these vaccines diminishes after several months, requiring additional booster doses for sustained protection [[23], [24], [25], [26]]. Concerns also have been raised regarding the increased risk of thrombotic events, known as Vaccine-induced Immune Thrombocytopenia (VIT), associated with developed adenoviral vaccines [27]. Most of the reported mutations are noticed in the RBD of the emerged SARS-CoV-2 strains due to which the developed vaccines are found to be ineffective [9,18,28]. Reports also indicate that the titers of the neutralizing anti-RBD antibodies decay at a faster rate [16,22]. However, the ongoing discussions about the side effects of a SARS-CoV-2 vaccine (AstraZeneca) and the circulating new strains of SARS-CoV-2, FLiRT (KP .2 & JN .1) to which none of the developed vaccines are effective, certainly support the need for better vaccines for this virus.

## 2 Materials and methods

### 2.1 Identification of epitopes

The protein sequence of the spike protein of SARS-CoV-2 from India, accession no. BCN86353.1, was obtained from the NCBI database. To predict the accurate linear B-cell epitopes, the Immune Epitope Database (IEDB) resource was used [29]. The above method predicts specific regions of the spike protein being on the surface, immunogenic, present in the hydrophilic region, containing beta turns and that binds to the B-cell receptor [[30], [31], [32], [33]]**.** Potentially continuous and discontinuous B cell epitopes were predicted using Ellipro from the IEDB resource [34]. From the obtained data, the B cell epitopes of the spike protein present in the non-RBD region and on the surface were chosen.

### 2.2 Display of epitopes on protein structure

The 3D structure of the spike protein was obtained from the Research Collaboratory for Structural Bioinformatics (RCSB) database and displayed using the Molecular Display Programme, PyMol [35]. The predicted B-cell epitopes are highlighted on the displayed structure [36].

### 2.3 Antigenicity analysis

The antigenicity of the identified epitopes, spike protein, and its RBD was determined by using the VaxiJen v 2.0 server (http://www.ddg-pharmfac.net/vaxijen/VaxiJen/VaxiJen.html) with the default parameters applicable for viruses (threshold = 0.4) [37].

### 2.4 Conservancy, allergenicity, and toxicity analyses of epitopes

The retrieved amino acid sequences of spike glycoprotein from major SARS-CoV-2 strains were aligned using the ClustalW algorithm of the BioEdit software version 7.2.5 to identify the conserved regions within the sequences [38,39]. The allergenicity of the predicted epitopes was analyzed by using the AllerTop tool (https://www.ddg-pharmfac.net/AllerTOP/) [40]. Toxin Pred server (http://crdd.osdd.net/raghava/toxinpred/) was used to predict the toxicity assessment of epitopes [41].

### 2.5 Homology with the human sequence

The homology of the predicted epitope sequences with the human sequences was analyzed using protein blast analysis tool (https://blast.ncbi.nlm.nih.gov/Blast.cgi?PROGRAM1⁄4blastp&PAGE_TYPE1⁄4BlastSearch&BLAST_SPEC1⁄4&LINK_LOC1⁄4blasttab). [42].

### 2.6 Prediction of cytotoxic T-cell epitopes

Cytotoxic T-cell epitopes were predicted by the NeTCTL1.2 server [43]. The spike protein from SARS-CoV-2 was analyzed using the IEDB MHC-I binding prediction tool to predict the T-cell epitopes interacting with different types of MHC class I alleles. The parameter was set at 50 to have the highest specificity and sensitivity of 0.94 and 0.89, respectively. MHC class I binding T-cell epitopes were predicted by the IEDB using the stabilised matrix method (SMM), and the epitopes binding to HLA alleles at scores equal to or less than 1.0 percentile rank were selected. A reference panel of 27 alleles recommended by the IEDB was selected. Further, the CTL epitopes were cross checked for their immunogenicity using the IEDB MHC I immunogenicity prediction tool [44]**.**

### 2.7 Prediction of helper T-cell epitopes

The IEDB NetMHCIIpan tool was used to predict 9-mer helper T-cell binding epitopes of spike protein. The analysis of epitopes binding to MHC class II molecules was performed using the SMM-based NetMHCIIpan 3.0 server, which covers all alleles of HLA-DR, HLA-DQ, and HLA-DP. The epitopes with low IC50 values suggesting good MHC binders were selected [45].

### 2.8 Human subjects involved in the study

The study included a total of 12 participants (Suppl Table 3). At the time of the study, seven people had a history of COVID-19 but no symptoms. There were five vaccinated (three with Covishield and two with Covaxin), but no history of SARS-CoV-2 infection. Informed consent was obtained before the initiation of the study. Approximately 5 ml of blood was collected from each participant.

### 2.9 Procurement of the epitopes

Epitope I (aa 80–90) and epitope II (aa 262–270) were synthesized commercially (Biotech desk Pvt. Ltd; Hyderabad, India). The small protein (aa 1059–1124) consisting of 65 amino acids with 3 epitopes (aa 1063–1068, 1069–1080, and 1114–1123) was cloned into a pRSET-A vector and expressed in-house. For this purpose, the nucleotide sequence coding the above small protein was PCR amplified using the full-length spike protein sequence (Addgene, USA, Cat. No. 145037) as the template. The primers used were forward 5′ GCGCGGATCCATGGGCTATCATCTTATGTCCTTC 3′ and reverse 5′ CGTGCGGGAATTCGGTGTTTAGTAATGATGTC 3′, with the 5′ *Bam*HI and 3’ *Eco*RI restriction enzyme sites. The PCR conditions applied were 95°C-5 minutes of initial denaturation, 35 cycles of PCR (95°C-30 s, 56°C-30 s, 72°C-30 s) followed by 72°C-5 minutes of final extension. The amplified and restriction enzyme digested insert was ligate to similarly digested vector (pRSET-A) and transformed into *E. coli* DH5 a cells. The suspected colonies were screened and positive recombinants were used in subsequent studies.

### 2.10 Expression and purification of small protein

The small protein containing PRSET-A recombinant plasmid was transformed into *E. coli* BL21 DE3 cells. The obtained colonies were grown in 500 ml of Luria broth (LB) containing 100 mg/ml ampicillin at 37 °C until the cultures reached 0.6 OD and induced with 0. 5 mM IPTG. The overexpressed protein was purified using Ni-NTA affinity column chromatography.

### 2.11 Expression of SARS-CoV-2 spike protein

The recombinant construct PGBW–spike was transformed into *E. coli.* BL21 DE3 cells, obtained colonies were inoculated into 50 ml of Luria broth (LB) medium containing 40 μg/ml chloramphenicol and grown at 30 °C until the OD reached 0.6. Then the expression was induced by IPTG at 0.5 mM at 18 °C for 18 h. The obtained cell lysate was subjected to sonication, and the supernatant was used for further studies.

### 2.12 Antibody raising

Three month aged rabbit was immunized intramuscularly with 150 ml of phosphate buffered saline (PBS) containing 10 μg of small protein emulsified with 150 ml Freund's complete adjuvant, followed by three booster immunizations with Freund's incomplete adjuvant. After seven days of the fourth dose, the animal was bled to collect immune serum. Epitopes I and II were mixed in equal parts, and antibodies were raised as above using a different rabbit.

### 2.13 Titer determination

A 96-well ELISA plate was coated with small protein (100 ng/well) in carbonate buffer pH 9.6 and incubated overnight at 4 °C. After washing and blocking, 100 ml of different pre-immune and anti-serum dilutions (1:250, 1:500, 1:1000, 1:2500, 1:5000, and 1:10,000) were added. After washing, goat anti-rabbit IgG HRP conjugated secondary antibodies (1:2000) (Santa Cruz; Catalogue no. Sc 2004) were added and incubated for 1 h at 37 °C. The developed reaction (OD) was measured after adding the substrate and a graph was plotted using the OD and the serum dilutions. The titer was calculated as the last dilution with an OD value twice that of the negative control (pre-immune serum). A similar protocol was used to determine the titer of the antiserum of the epitope mix.

### 2.14 Western blot immunoassays

*E. coli* BL21 cell lysate expressing spike protein and purified small protein were electrophoresed on 10 % and 12 % SDS-PAGE, respectively, and transferred onto the separate polyvinylidene fluoride (PVDF) membranes for immunoblotting. The membranes containing spike protein/purified small protein were probed with antisera of epitope mix (1:5000), small protein (1:2500), and COVID-19 recovered’ (1:5000) dilutions for overnight at 4 °C. Secondary HRP-conjugated anti-rabbit and anti-human IgG antibodies (Santa Cruz; Cat. No. Sc 2907) were used at 1:10,000. The developed images after adding the femto LUCENT PLUS HRP substrate (G- Bioscience, Cat No. 786–003) reagent were recorded using the Gel Doc imager (Bio-Rad).

### 2.15 Complement fixation test (CFT)

Before performing the test, the sera (anti-peptide mix, anti-small protein and COVID-19 recovered) were diluted to 1:50 and incubated at 60 °C for 30 min to inactivate the native complement. In 1.5 ml Eppendorf tubes, 25 ml of the aforementioned sera were mixed with antigen. Then, 25 ml of Guinea pig complement (Medaux-Bio, Cat. No. MX-1011-01) was added and incubated at 37 °C for 4 h. Sensitized sheep RBCs (pre-coated anti-sRBCs) were added and incubated at 37 °C for 60 min, then centrifuged at 200 g for 5 min to allow the unlysed RBCs to deposit. The positive reaction (clump or pellet) suggests the fixing of the complement by the antigen-antibody complex. In the negative control, unbound complement lysed the sensitized RBCs (hemolysis).

### 2.16 Activation of SARS-CoV-2 exposed/vaccinated PBMCs (memory cells) by the epitopes

PBMCs were isolated from 4 ml of freshly drawn blood from COVID-19 recovered and vaccinated individuals using the standard Ficoll-histopaque (Sigma Aldrich; Cat. No. 11191) density gradient centrifugation method. Heparin-treated fresh blood was diluted in PBS at an equal volume. 5 ml of ficoll was added slowly to a 15-ml centrifuge tube, followed by the addition of 8 ml of diluted blood. Centrifugation was carried out at 250 g, 22 °C for 20 min. The mononuclear cells at the interface were collected and washed twice with PBS. A total of 1 × 10^6^ cells were resuspended in RPMI 1640 medium (Gibco BRL; Cat. No. 11-875-093) to which 10 % fetal bovine serum (FBS) (Gibco BRL; Cat. No. 26140079) was added and seeded. Then the stimulation of PBMCs was done with 10 mg/ml of epitope mix and small protein. Phorbol myristate acetate (PMA) (100 ng/ml) (Sigma; Cat. No. 16561-29-8) was used as a positive control for cytokine activation. PBMCs treated with only PBS were considered a negative control. After 48 h of stimulation, the cells were incubated for 6–8 h with Brefeldin A (25 ng/ml) (Thermo Fisher; Cat. No. 00-4506-51) to inhibit the cytokine secretion. Then the cells were simultaneously stained for cytochrome-conjugated anti-CD3^+^ (APC Cy7), anti-CD4^+^ (PE Cy7), and anti-CD8^+^ (APC) surface markers. The cells were permeabilized, fixed, and stained for intracellular markers IL-2 (PE-Biolegend; Cat. No. 500306) and IFN-g (FITC-Biolegend; Cat. No. 502505). The stained cells were analyzed by flow cytometer (BD Biosciences Fortessa). Gates on physical parameters and cytochrome staining were integrated for the measurement of cell surface and intracellular cytokine staining. Dot plots using Flowjo software (BD Biosciences) were used to calculate the percentage of IL-2 and IFN-g secreting T cells. The levels of IL-2 and IFN-g were graphed as arithmetic means with standard deviation in stimulated cells along with positive and negative controls. One-way ANOVA statistical tests were performed using GraphPad Prism Version 6.01 (GraphPad Software, San Diego, California, USA).

## 3 Results

### 3.1 The epitopes are located at the non-RBD and on the surface of spike protein

**Fig. 1:**
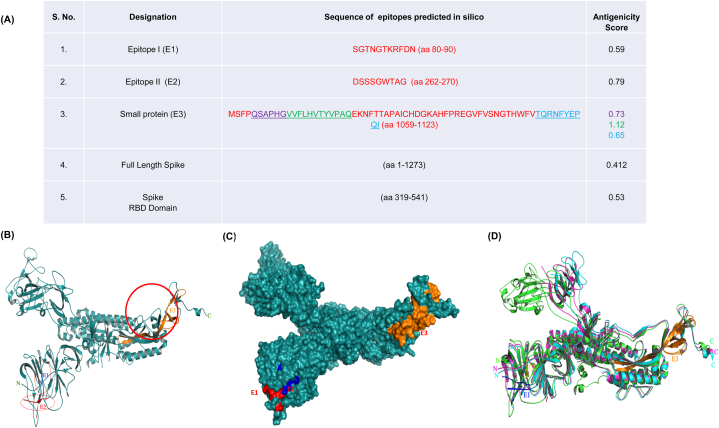
**Display of the epitopes on spike protein structure**. (A) Epitope sequences and their antigenicity scores (S. Nos 1, 2 and 3-epitopes of the present study). Epitope I (aa 80–90), Epitope II (262–269), small protein (1059–1123) are located as on SARS-CoV-2 spike protein. (B) 3D structure of spike protein ‘A’ chain representing epitope location. The blue (epitope I), red (epitope II) & orange (small protein) coloured regions represent the epitopes predicted (epitopes I&II are highlighted in circles). (C) Surface representation of epitopes on spike protein ‘A’ chain: EI-red; EII-blue; EIII-orange. (D) Superimposed 3 D structures of spike protein ‘A’ chain of three different strains of SARS-CoV-2: Green- COVID-19 Native type, Pink – Omicron, Cyan-Delta. N and C represent the N- and C-terminal ends, respectively.

Reports regarding the developed SARS-CoV-2 vaccines discourage the use of complete spike protein and its RBD [15,28]. Hence, this study focused to identify the epitopes with two conditions. They were 1. The epitopes should be of non-RBD region and 2. They should be located on the surface of the protein because protective antibodies target epitopes that lie on the surface of the pathogen. In this direction, the epitopes that have been identified are epitope-I (aa 80–90), epitope-II (aa 262–270) in NTD, and a small protein with three epitopes (aa 1063–1068, 1069–1080, and 1114–1123) in heptapeptide region of spike protein (Fig. 1 A). The numbering of amino acids (aa) is according to the amino acids of spike protein.

The conformational B-cell epitopes were obtained on the ‘A’ chain of the spike protein by using the Ellipro software. The highest probability of a conformational epitope was calculated at 86 %. In epitopes I & II, the number of residues that can be potential B cell epitopes is 5 and 7, respectively (PI score 0.72), and in small protein 15 amino acids have the highest score (0.96) (Table 1). Spike protein was subjected to Bepipred linear epitope prediction, in which the average binding score of spike protein to B-cell was −0.066, with a maximum of 2.291 and a minimum of −0.001. The epitopes with values equal to or greater than the default threshold (0.35) were predicted to be the potential B-cell binders. In Emini surface accessibility prediction, the average surface accessibility area of the protein was scored as 1.0, with a maximum of 6.051 and a minimum of 0.042. The epitopes identified in this study were on the surface with values equal to or greater than the default threshold (1.0). The protein's antigenicity threshold was set at 1.041, with a maximum of 1.261 and a minimum of 0.866. The Chou and Fasman beta turn prediction method was used to detect epitope confirmation with a threshold of 0.997, a maximum of 1.484, and a minimum of 0.54. The data suggests that the small protein is in the beta turn helix and flexible in nature, indicating that the epitopes of the small protein could interact with the neutralizing antibodies.

**Table 1:** Characteristics of B-cell Epitopes analyzed using Ellipro tool.

Designation	Residues	No. of residues	PI Score
Epitope I	A∗:K86, A:R87, A:F88, A:D89, A:N90	5	0.728
Epitope II	A∗:S263, A:S264, A:G266, A:W267, A:T268, A:A269, A:G270	7	0.728
Small Protein	A∗:A1080, A:I1081, A:C1082, A:H1083, A:D1084, A:G1085, A:K1086, A:A1087, A:K1088, A:W1089, A:G1099, A:T1100, A:H1101, A:I1114, A:I1115	15	0.9

A∗-Amino acid Residues of ‘A’ chain of spike protein.

The three dimensional structure of the spike protein of SARS-CoV-2 was obtained from the RCSB database and the structure of the ‘A’ chain was displayed. This protein showed a good model with SWISS-MODEL by using PDB ID: 7DDD as a template, having 95.68 % identity and 100 % similarity with the query structure. In the structure, the predicted epitopes E1 & E2 are located near the flexible loops at the N-terminal domain of the spike protein (Fig. 1B; E1-blue; E2 -red & E3-orange). All five epitopes selected for this study were present on the surface (Fig. 1C). The ‘A’ chain structures were superimposed to determine the conformational differences, if any, among different variants of the coronavirus spike protein. The structural superimposition did not show any changes around the selected epitopes (Fig. 1D).

E1 and E2 are shorter in length and present at long distances, i.e., one at the 80th amino acid (epitope I) and the other at the 262 amino acid (epitope II). Hence, these two epitopes were considered to be synthesized separately for this study. The C-terminal domain (right side) appears to be important in maintaining the structural integrity of the protein. In this region, three epitopes of E3 encompassing amino acids 1063–1068, 1069–1080, and 1114–1123 were identified. Since the first and second epitopes of E3 are adjacent to each other and the third epitope is present at a few amino acid gaps, a sequence from amino acid 1059 to 1123 of the heptapeptide domain of S2 subunit of spike was considered a small protein (total 65 amino acids). This small protein was cloned, expressed, and purified.

### 3.2 The epitopes possess higher antigenicity

The antigenicity scores of epitopes I and II were found to be 0.79 and 0.59, respectively. The antigenicity scores of the epitopes identified in small proteins were predicted to be 0.73 for the epitope aa1063-1068, 1.12 for aa 1069–1080, and 0.65 for the epitope aa1114-1123. The antigenicity scores of identified epitopes were found to be higher than those of the full-length spike protein (0.412) and its RBD region (0.53) (Fig. 1 A).

### 3.3 The epitopes are highly conserved across the SARS-CoV-2 strains

**Fig. 2:**
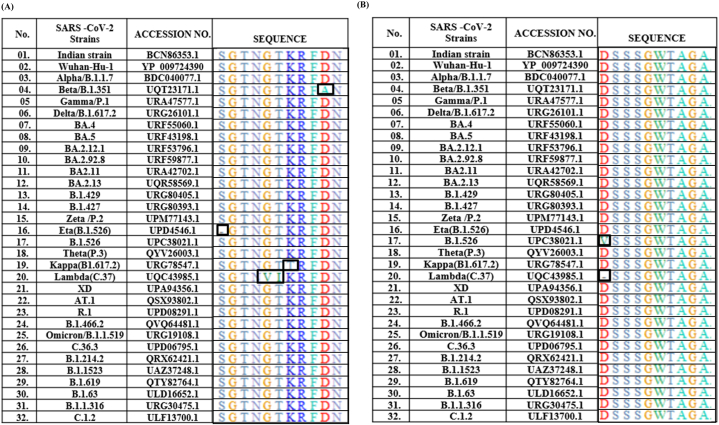

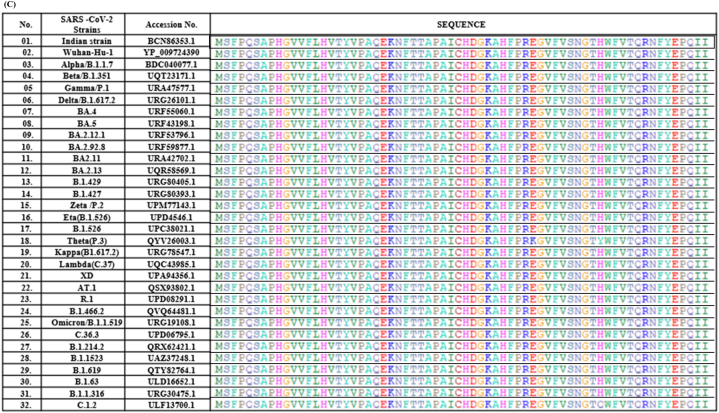
**Alignment of amino acid sequences of predicted epitopes from all major SARS-CoV-2 strains**. (A) Epitope I show 1 amino acid substitution in each of two strains and 2 in one strain (Beta B.1.351 - D89A; Kappa B.1.617.2-K86T; Lambda C.37 G84V, T85I), one deletion (Eta B.1.526 -S80). (B) Epitope II shows 1 substitution (Eta B.1.526 -D262V) and one deletion (Lambda C.37- D262). The substitutions/deletions are indicated in squares. (C) Small protein shows 100 % conservancy in the epitopes of all strains.

The BioEdit software alignment suggests that there is one amino acid substitution in the SARS-CoV-2 strain: Beta/B.1.351, Kappa/B.1.617.2, two amino acid substitutions in Lambda/C.37, and one deletion in the strain Eta/B.1.617.2; in the case of epitope I (Fig. 2A). There is one amino acid substitution (B.1.526) and one deletion (Lambda/C.37) in the case of epitope II (Fig. 2B). Sequence alignment of the small protein of all major SARS-CoV-2 strains shows 100 % conservancy in all three epitopes (Fig. 2C). The epitopes were found to be non-allergic and non-toxic in nature (Suppl Tables 1 and 2).

### 3.4 Epitopes show no homology with the human sequences

In protein blast analysis, none of these epitope sequences were found to be homologous to human sequences.

### 3.5 Epitopes show higher affinity for MHC I & II binding

Using NeTCTL and SMM based IEDB MHC-I binding prediction tools, the S protein epitopes that interacted with different MHC-I alleles with higher affinity (IC50 less than 200 nm) were selected based on the predicted proteasome, TAP, and MHC-I binding scores (Suppl Table 1). Similarly, the epitopes with IC50 values less than 200 nm were found to have a higher affinity for MHC II binding (Suppl Table 2).

### 3.6 Small protein containing 3 epitopes was expressed and purified

Recombinant pRSET-A construct containing small protein was generated and confirmed (Suppl Fig. 1 A). *E. coli* BL21 DE3 cells were transformed with the above construct and induced for its expression. The purification of the expressed protein was performed by Ni-NTA affinity chromatography. The small protein was eluted at an imidazole concentration of 250 mM. The size and purity of the eluted protein were analyzed using 12 % SDS PAGE, which showed the migration at ∼15 kDa (Suppl Fig. 1 B, lanes E3 & E4).

### 3.7 Epitopes induced good antibody titer

Before immunizing the rabbit, 5 ml of blood was collected as pre-immune serum. After 7 days of immunization, ∼5 ml of blood was collected for preliminary analysis. After the final injection, the blood was collected and the serum was separated; glycerol was added to 15 % and stored at −20 °C for long term usage.

To determine the titer of antibodies raised against the small protein and epitope mix, an indirect ELISA method was carried out. The last dilution with an OD of twice that of the negative control was considered the titer of the antibody. Epitope mix and small protein antibody titers were found to be 1:5000 and 1:2500, respectively (Suppl. Fig. 2).

### 3.8 Anti-epitope antibodies interacted with the SARS-CoV-2 spike protein

**Fig. 3:**
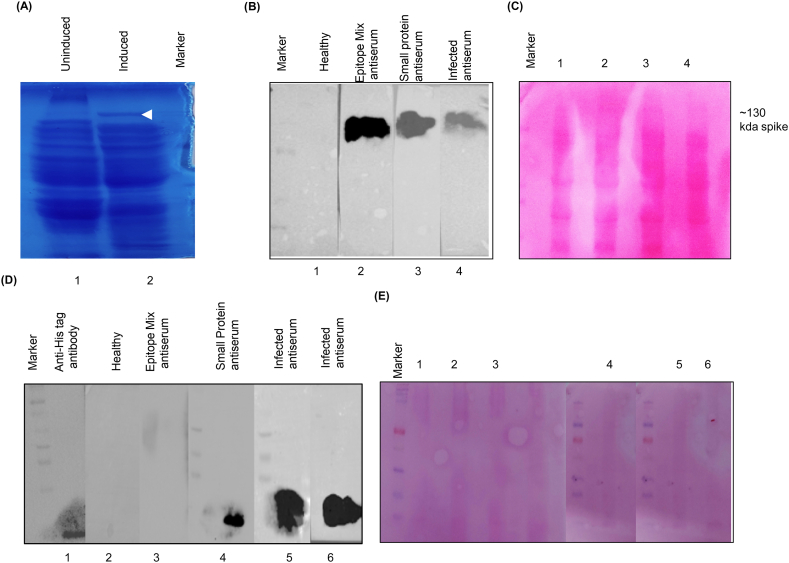
**Western blotting analysis of the identified epitopes**. (A) 10 % SDS-PAGE resolution of bacterial cell lysate (arrow indicated spike protein). (B) Full-length spike protein probed with indicated primary antibodies on the blot image, (C) Ponceau staining of the same membrane. (D) Small protein probed with the primary antibodies indicated on the blot image, (E) Corresponding Ponceau S-stained PVDF membrane. (The original images are provided in the supplementary file).

The bacterial lysate expressing S protein was analyzed on 10 % SDS-PAGE and the S protein was found to resolve at approximately 130 kDa (Fig. 3A, lane 2). The western blotting analysis for the above lysate suggest that the antibodies raised against epitope mix, small protein, and recovered serum (Suppl Tables 3 and S. NOs. 1& 2) were found to interact with the spike protein (Fig. 3 B, lane 2–4). Similarly, in the case of purified small protein the data suggest that the small protein interacted with the homologous antibody raised against it and the antibodies of the recovered serum (Fig. 3 D, lane 4–6). The small protein did not interacted with the antibodies of epitope mix I & II as their location is different (Fig. 3 D, lane 3).

### 3.9 The epitopes and the homologous/convalescent antibodies successfully fixed the complement

**Fig. 4:**
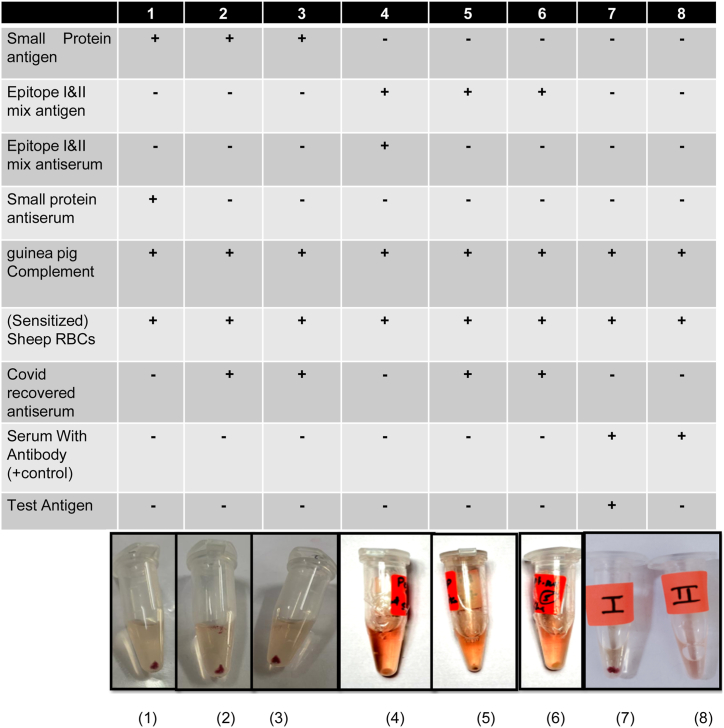
**Complement Fixation Test (CFT)**: Epitope I&II mix and small protein as antigens were used against their homologous antibodies and antibodies present in COVID-19 recovered serum. The reagents used are indicated on each tube presented in the image. Complement fixation was observed in lanes 1–6, lane 7 is a positive control. Lane 8 with hemolysis is negative control.

The complement fixation test was performed on sera collected from immunized rabbits with an epitope mix, small protein, sera collected from COVID-19 recovered and vaccinated participants. The reaction components are indicated on each tube of the experiment (Fig. 4). Complement fixation was observed with epitope mix and small protein as the antigens (Fig. 4, lanes 1–6) along with the positive control (lane 7). In the negative control, hemolysis was observed in the absence of the above epitopes or their compatible antisera (Fig. 4, lane 8).

The epitopes successfully fixed the complement in the presence of the self anti-serum, sera collected from COVID-19-recovered and vaccinated participants.

### 3.10 The epitopes induced the memory T-cells efficiently

**Fig. 5:**
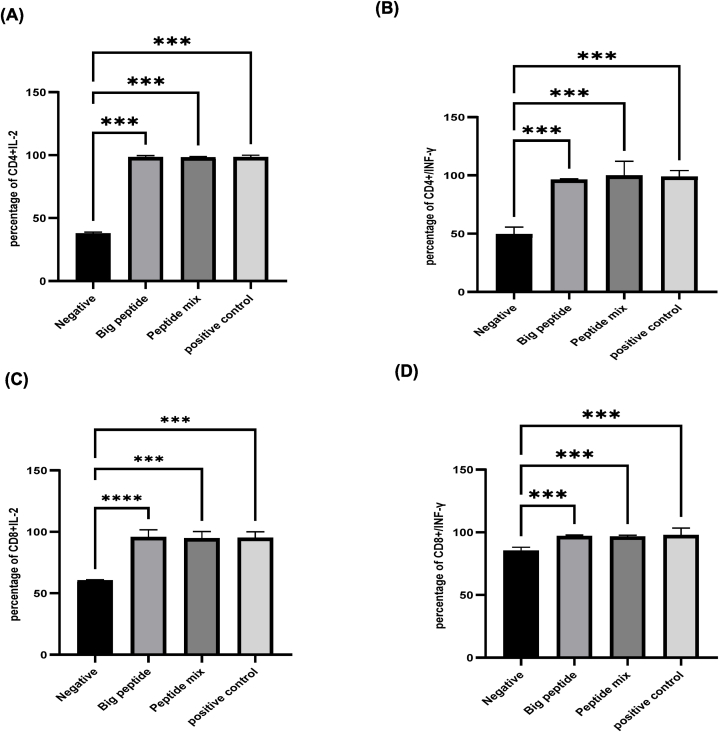
**Bar diagram representation of IL2 and IFN- γ secreting CD4**^**+**^**and CD8**^**+**^**T cells from PBMCs of COVID-19 recovered samples**. Data are shown as mean ± SEM. Statistical tests performed by one way ANOVA Test, n = 3, P value 0.0001. (A) Percentage of IL2 secreting CD4^+^ T cells, (B) Percentage of IFN-γ secreting CD4+T cells, (C) Percentage of IL2 secreting CD8^+^ T cells, (D) Percentage of IFN-γ secreting CD8^+^ T cells.

Out of the total 12 PBMC isolated samples, seven were COVID-19 exposed and recovered; five were vaccinated (three with Covishield and two with Covaxin) (Suppl Table 3). The data indicated that CD4^+^ and CD8^+^ cell proliferation was significantly increased upon activation by the epitopes of the present study (Suppl. Figs. 3 and 4; Table 2A & Table 2B). The activated CD4^+^ and CD8^+^ cells were found to secrete significantly higher levels of IL-2 and IFN-g cytokines (Fig. 5 and Suppl Fig. 5).

**Table 2A:** % Increase in CD+4 (IL2 & IFN gamma secreting) cells over the negative control.

	Covid 19 recovered PBMCs		Covishield vaccinated PBMCs		Covaxin vaccinated PBMCs	
	**Peptide Mix**	**Small Protein**	**Peptide Mix**	**Small Protein**	**Peptide Mix**	**Small protein**
**IL2**	**64**	**58**	**45**	**50**	**30**	**45**
**IFN gamma**	**56**	**53**	**49**	**40**	**25**	**35**

**Table 2B:** % Increase in CD+8 (IL2&IFN gamma secreting) cells over the negative control.

	Covid 19 recovered PBMCs		Covishield vaccinated PBMCs		Covaxin vaccinated PBMCs	
	**Peptide Mix**	**Small Protein**	**Peptide Mix**	**Small Protein**	**Peptide Mix**	**Small protein**
**IL2**	**76**	**80**	**30**	**41**	**20**	**25**
**IFN gamma**	**51**	**47**	**54**	**52**	**25**	**20**

The CD4^+^ and CD8^+^ responses were compared in the PBMCs of COVID-19 recovered and vaccinated as follows:

#### 3.10.1 CD4^+^ response

In PBMCs of COVID-19 recovered, the percentage of IL2 and IFN-γ secreting CD4^+^ T cells was enhanced by 64 % and 56 % if activated with epitope mix [Fig. 5 A& B; Table 2 A; Suppl Fig 3 B (d&e)]. Similarly, there was a 58 % and 53 % increase in IL2 and IFN-γ secreting CD4^+^ cells if activated with small protein [ Fig. 5 A&B; Table 2 A; Suppl Fig 3C (d&e)]. In the case of Covishield vaccinated PBMCs, the IL2 and IFN-g secreting CD4^+^ cells were found to be 45 % and 49 % higher in epitope mix activated; 50 % and 40 % higher in small protein induced cells [Suppl Figs 4 B (d&e); 4C (d&e); 5 A&B; Table 2 A]. In Covaxin vaccinated, the percentage of IL2 and IFN-g secreting CD4^+^ cells was 30 % and 25 % respectively, with the epitope mix; 45 % and 35 % higher in the case of small protein (Table 2 A).

#### 3.10.2 CD8^+^ response

In COVID-19 recovered PBMCs, the percentage of IL2 and IFN-g secreting CD8^+^ T cells was enhanced by 76 % and 51 % activated with epitope mix; 80 % and 47 % higher if activated with small protein compared to the negative control [Suppl Fig 3 B (f&g) & C (f&g); Fig. 5C and D; Table 2 B]. The percentage of IL2 and IFN- g secreting CD8^+^ cells was also increased in the PBMCs isolated from Covishield vaccinated and treated with peptide mix (IL2 by 30 %, IFN-g by 54 %) and small protein (IL2 by 41 %, IFN-g by 52 %) [Suppl Fig. 4B (f&g) & C (f&g) 5C & D]. Similarly, in case of the Covaxin, the memory cells were found to be activated, if treated with peptide mix (IL2 by 20 %, IFN-g by 25 %) and small protein (IL2 by 25 %, IFN-g by 20 %) (Table 2 B).

The data suggested that the activation of the memory cells (PBMCs) by the epitopes was less in the case of vaccinated compared to the PBMCs of COVID-19 recovered samples.

## 4 Discussion

COVID-19 caused millions of cases of infections and deaths worldwide, and the trend is continuing today. Scientists across the world made efforts to understand the virus characteristics and develop control measures at the earliest possible time. In this direction, the vaccine candidates developed represent all the vaccine development approaches established until now [13]. But every vaccine candidate shows some issues with reference to safety, efficacy, long-lasting memory, etc. Epitope identification is critical in vaccine design and development. Common vaccines induce polyepitopic responses with anti-pathogen properties, but they may also induce “non-neutralizing enhancing” antibodies. Such antibodies facilitate infection, as occurs in several viruses including SARS-CoVs, or even recently shown in SARS-CoV-2 [[15], [16], [17]]. This issue can be solved by using the peptide based subunit vaccines that employ only immunogenic environment exposed sequences which produce non-inflammatory neutralizing or opsonic immune responses [18,46]. B and T lymphocytes usually recognize small epitope regions of the antigens. Naturally occurring antibodies, or T cell receptors are being used as templates for vaccine design. Because a particular epitope that already triggered a B or T cell response during a natural infection is expected to induce similar responses if administered as a vaccine candidate [15]. Identification of such epitopes is the basis for the development of peptide vaccines. Studies have shown that T memory cells recognize the epitopes of certain SARS-CoV-2 proteins from COVID-19 convalescent patients [47,48]. On the other hand, many attempts have been made using immunoinformatic tools to identify potential SARS-CoV-2 epitopes, while only a few synthetic peptides have been tested as vaccine candidates [49,50]. Peptide-based vaccines may confer some advantages over vaccines consisting of larger protein sequences or whole inactivated viruses, as they are smaller and may elicit a more focused immune response towards critical neutralizing epitopes [15]**.** They also avoid off-target antigen loss and combine antigens with different protective roles or mechanisms. Some practical features, such as their ease and flexibility of design, chemical synthesis and manufacturing, and their high stability, have also been considered as advantages for these immunogens.

A potential vaccine candidate is expected to elicit both innate and adaptive immune responses in order to yield timely and complete protection. Epitopes I and II of the present study are linear, and the small protein was a beta-strand in structure, which is required for efficient binding to the antibodies. These epitopes were found to be located on the surface of the protein (Fig. 1 B), suggesting easy access for antibody binding [51,52]. The structural integrity of the spike protein is expected to be blocked upon interaction with antibodies of small protein (E3), as this portion of the protein is involved in allowing the structural flexibility. The antigenicity of the epitopes was found to be higher than that of the spike protein as such and its RBD (Fig. 1 A) which is critical for inducing both innate and adaptive immune responses. The number of amino acid residues in each of these epitopes and their PI scores suggest that they are potential B cell epitopes (Table 1). These observations indicated the efficiency of the epitopes in eliciting the humoral immune response, which is further supported by the antibody titers (1:5000 and 1:2500 for epitope mix and small protein, respectively) (Suppl Fig. 2). There are several variants of SARS-CoV-2 reported ever since its emergence. Some of these variants were reported to be more virulent, escape the effect of the existing vaccines and have caused repeated outbreaks with high mortality. In this direction, the Omicron variant replaced the Delta and turned into the most prevalent SARS-CoV-2 variant across the world. This variant showed 37 mutations in total of which 15 are located on the RBD. These mutations could provide resistance to T cells, which are essential to target and destroy virus‐infected cells and could improve the capability to escape the immune system by affecting antibody recognition. As a consequence, it could alter the effectiveness of current vaccines [**9**, **28**]. Hence, we have analyzed the conserved nature of the identified epitopes across the emerging SARS-CoV-2 strains and found them to be highly conserved [Fig. 2]. This observation led to hypothesize that the antibodies raised against these epitopes would react with all the emerging SARS-CoV-2 strains until now and in future. Further, the anti-epitope antibodies reacted with the bacterially expressed full length spike protein in western blotting, suggesting the possibility of neutralization of spike protein in natural infections by the antibodies raised against these epitopes. The complement was also successfully fixed by these epitopes in the presence of self anti-sera and SARS-CoV-2 recovered participants, indicating that they are potential antigens capable of eliciting the innate immune response.

MHC-II T cell epitope complex activates CD4^+^ T cells, which are required for Th1 and Th2 immune responses. In this direction, CD4^+^ T cells activate CD8^+^ T cells, which gives rise to cytotoxic T-cells and memory T cells that can last for up to several years. As in the case of other infections, CD8^+^ T-cells are known to be potentially protective against SARS-CoV-2 infections also. This is true as in severe COVID-19 illnesses, the peripheral circulating CD8^+^ T-cell counts were found to be low, and hence the severity of illness is inversely correlated with CD8^+^ T-cell counts [18]. IFN-g is a cytokine that is produced primarily by natural killer (NK) and cytotoxic T lymphocytes (CTLs) and is involved in both innate and adaptive responses. According to reports, IFN-g plays a direct role in the inhibition of virus replication [53]**.** The studies demonstrate that distinct SARS-CoV-2 epitope peptide pools boosted the IFN-g response in the PBMCs of SARS-CoV-2 exposed patients [54]**.** On the other hand, IL2 promotes the differential proliferation of T cells into T regulatory, effector, and memory cells which is required for the elimination of the infections [55,56]. The PBMCs primed with the epitopes of the current study indicated that COVID-19 recovered and vaccinated PBMCs had a significant increase in the percent of CD4^+^ and CD8^+^ T cells with the increased IL2 and IFN- g secretions [(Fig. 5; Suppl Figs. 3,4 & 5, Table 2A, Table 2B A&B)]. Hence, it is concluded that the present study's epitopes can activate memory cells, which are the main component of adaptive immunity, and they can also fix the complement, which supports their function in innate immunity, suggesting that these epitopes cab be as promising vaccine candidates for SARS-CoV-2.

## CRediT authorship contribution statement

**Deepika Rathore:** Writing – original draft, Validation, Project administration, Methodology, Investigation. **Preeti Chauhan:** Writing – original draft, Methodology, Data curation. **Anvesh Bonagiri:** Writing – original draft, Validation, Methodology. **Lekha Gandhi:** Writing – original draft, Validation, Methodology, Formal analysis, Data curation. **Deepti Maisnam:** Writing – original draft, Validation, Methodology, Formal analysis, Data curation, Conceptualization. **Ramesh Kumar:** Methodology, Investigation, Data curation. **Anupama T. Row:** Methodology, Formal analysis, Data curation. **M.M. Kesavulu:** Writing – original draft, Validation, Methodology, Investigation, Data curation, Conceptualization. **Musturi Venkataramana:** Writing – original draft, Supervision, Resources, Project administration, Methodology, Investigation, Funding acquisition, Formal analysis, Conceptualization.

## Strengths and limitations

The strength of the present study is the ease of design of epitopes, cost effective and simple to test. As these epitopes are smaller, there will be no immunopathological effects and the anti-epitope anti-bodies are expected to target all the SARS-CoV-2 variants due to their high conserveness. Importantly, they have the potential to activate the memory cells. These features suggest them as promising vaccine candidates for SARS-CoV-2. On the other hand, the use of Epitope I & II as a mix to raise the antibodies is considered as the limitation of the study. Because currently, it is not known which among them is more immunogenic. Animal studies would have given more clarity, hence remains as another limitation of the study.

## IEC approval

The study was approved by the Institutional Ethics Committee, University of Hyderabad (UH/IEC/2022/350).

## Data availability

Data included in article/supp. material/referenced in article.

## Declaration of competing interest

The authors declare the following financial interests/personal relationships which may be considered as potential competing interests: Musturi Venkataramana reports administrative support and equipment, drugs, or supplies were provided by 10.13039/501100007741University of Hyderabad. Musturi Venkataramana reports a relationship with 10.13039/501100007741University of Hyderabad that includes: employment and non-financial support. Musturi Venkataramana has patent NA pending to NA. Nil.
