# Supplementary material for: Non-RBD peptides of SARS-CoV-2 spike protein exhibit immunodominance as they elicit both innate and adaptive immune responses
Source: Heliyon. 2024 Oct 29;10(21):e39941. doi: 10.1016/j.heliyon.2024.e39941 (PMC11577203; doi:10.1016/j.heliyon.2024.e39941)
Supplement: Multimedia component 1 [file mmc1.pptx]

## Slide 1
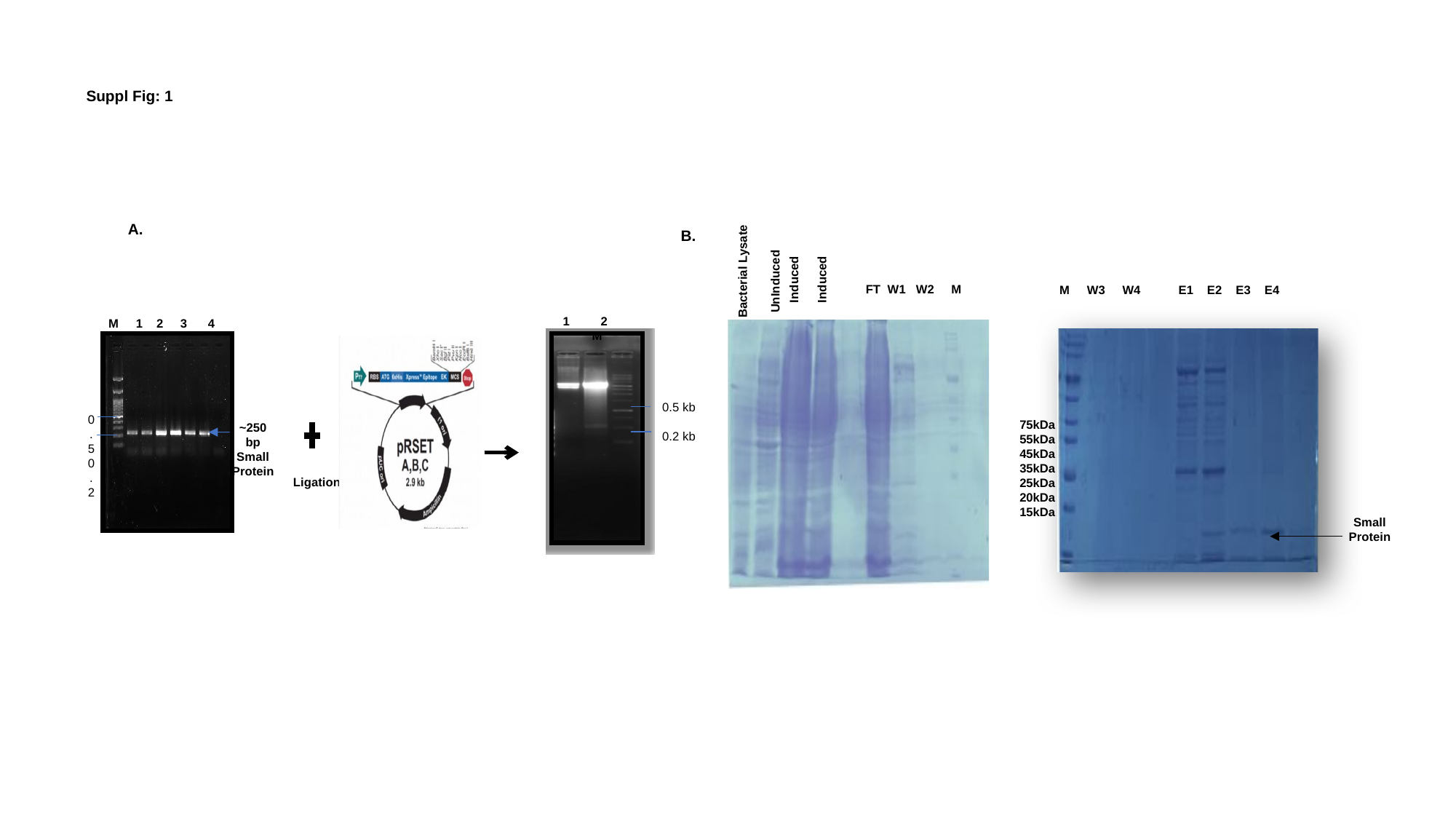

Suppl Fig: 1
Bacterial Lysate
Induced
Induced
UnInduced
FT W1 W2 M
M W3 W4 E1 E2 E3 E4
75kDa
55kDa
45kDa
35kDa
25kDa
20kDa
15kDa
Small Protein
A.
B.
 1 2 M
M 1 2 3 4 5 6
0.5 kb
0.2 kb
0.5
0.2
~250 bp Small Protein
Ligation

## Slide 2
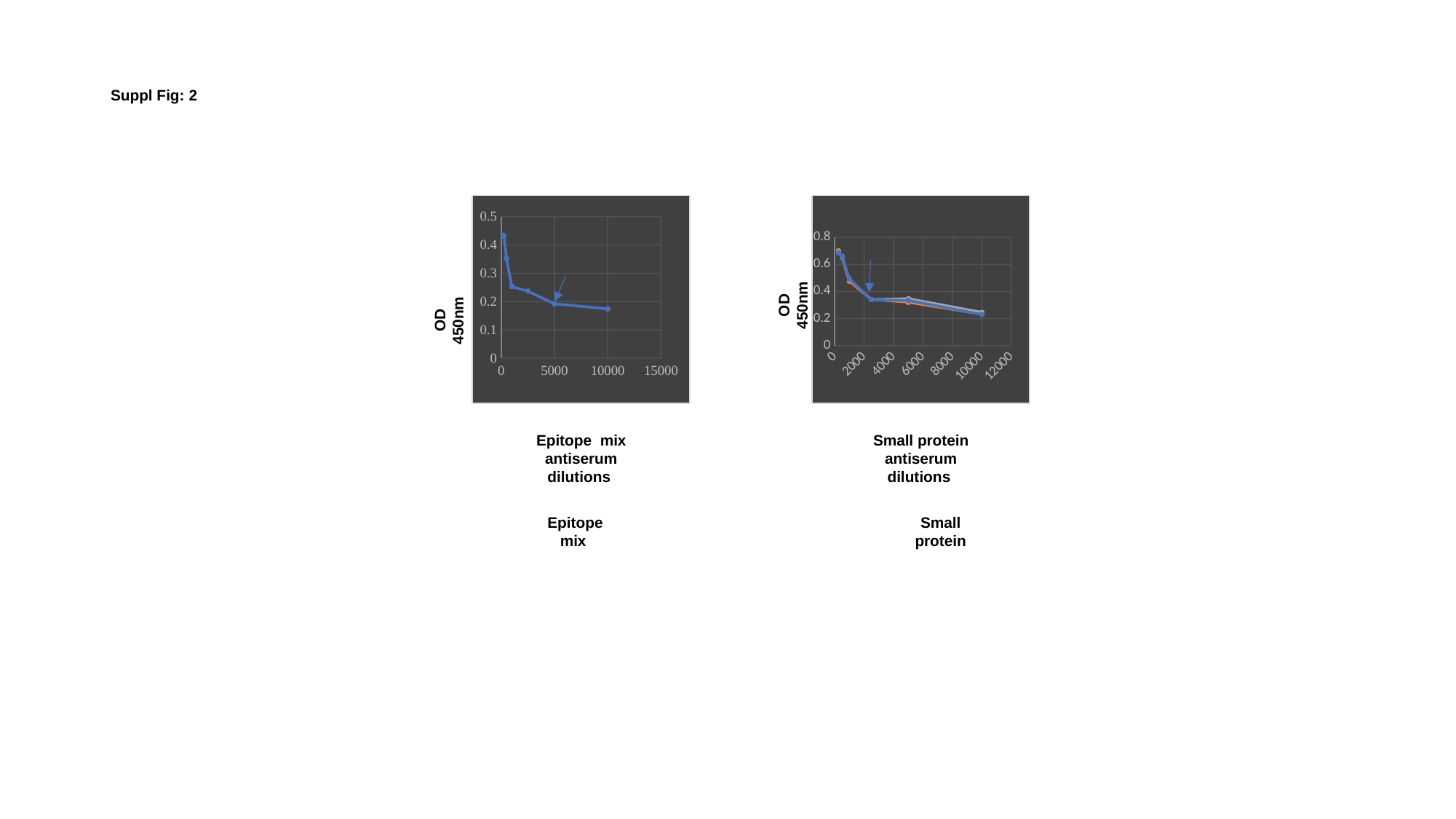

Suppl Fig: 2
### Chart
| Category | 0.167 |
|---|---|
### Chart
| Category | 0.183 | 0.183 | 0.208 |
|---|---|---|---|OD 450nm
OD 450nm
Small protein antiserum dilutions
Epitope mix antiserum dilutions
Small protein
Epitope mix

## Slide 3
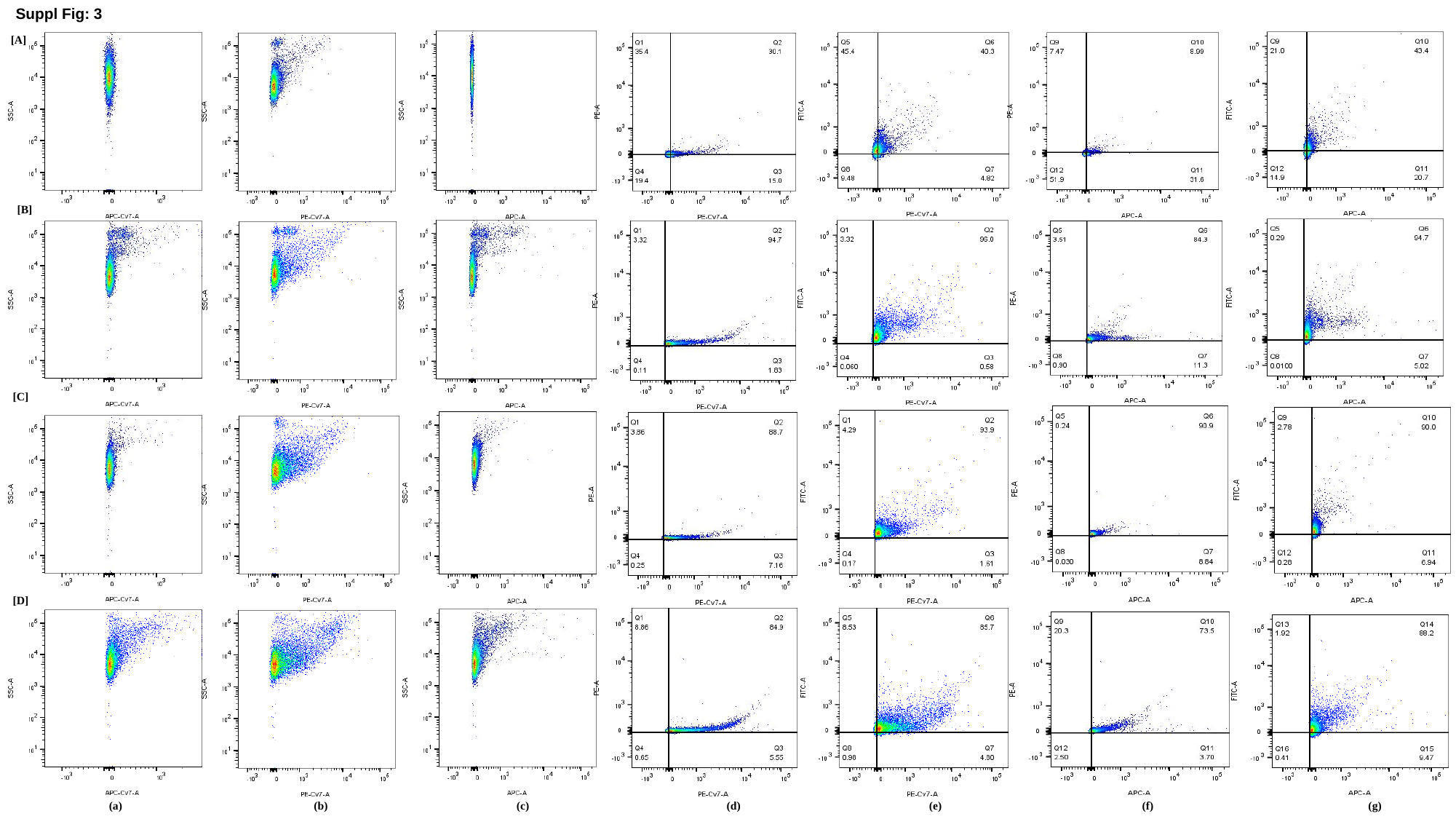

Suppl Fig: 3
[A]
[B]
[C]
[D]
(a) (b) (c) (d) (e) (f) (g)

## Slide 4
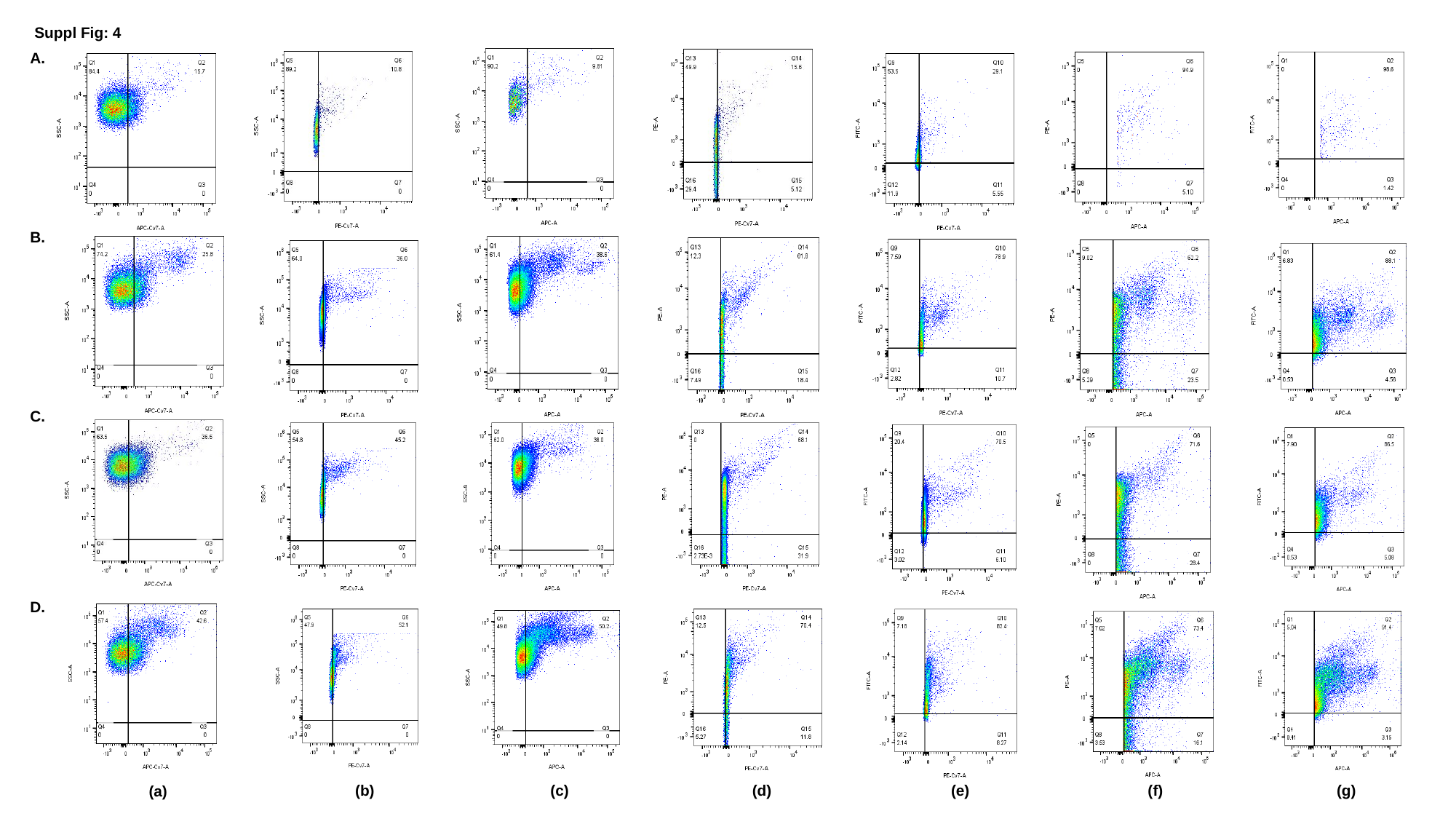

Suppl Fig: 4
A.
B.
C.
D.
(g)
(f)
(e)
(d)
(c)
(b)
(a)

## Slide 5
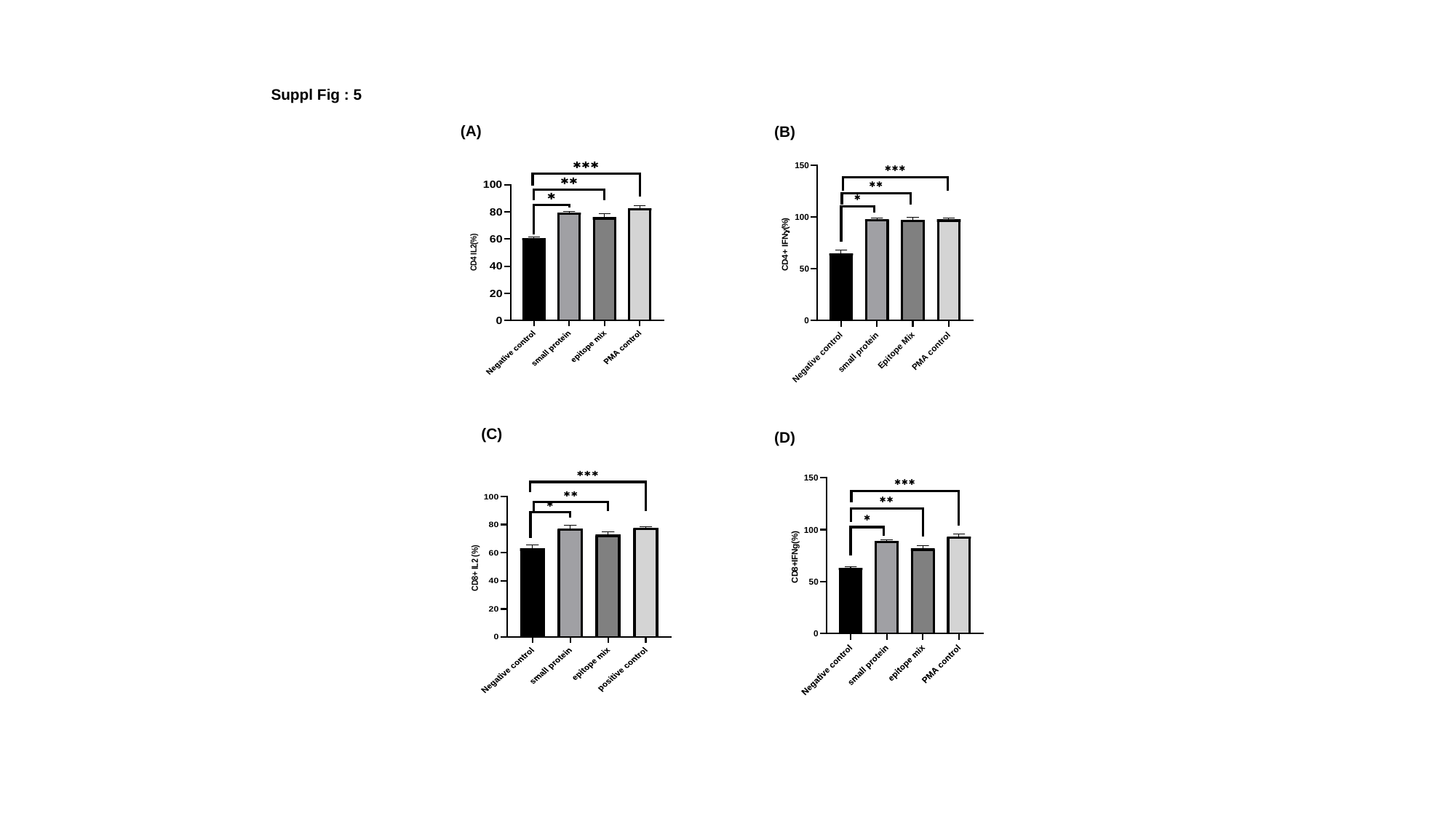

Suppl Fig : 5
(A)
(B)
(C)
(D)

## Slide 6
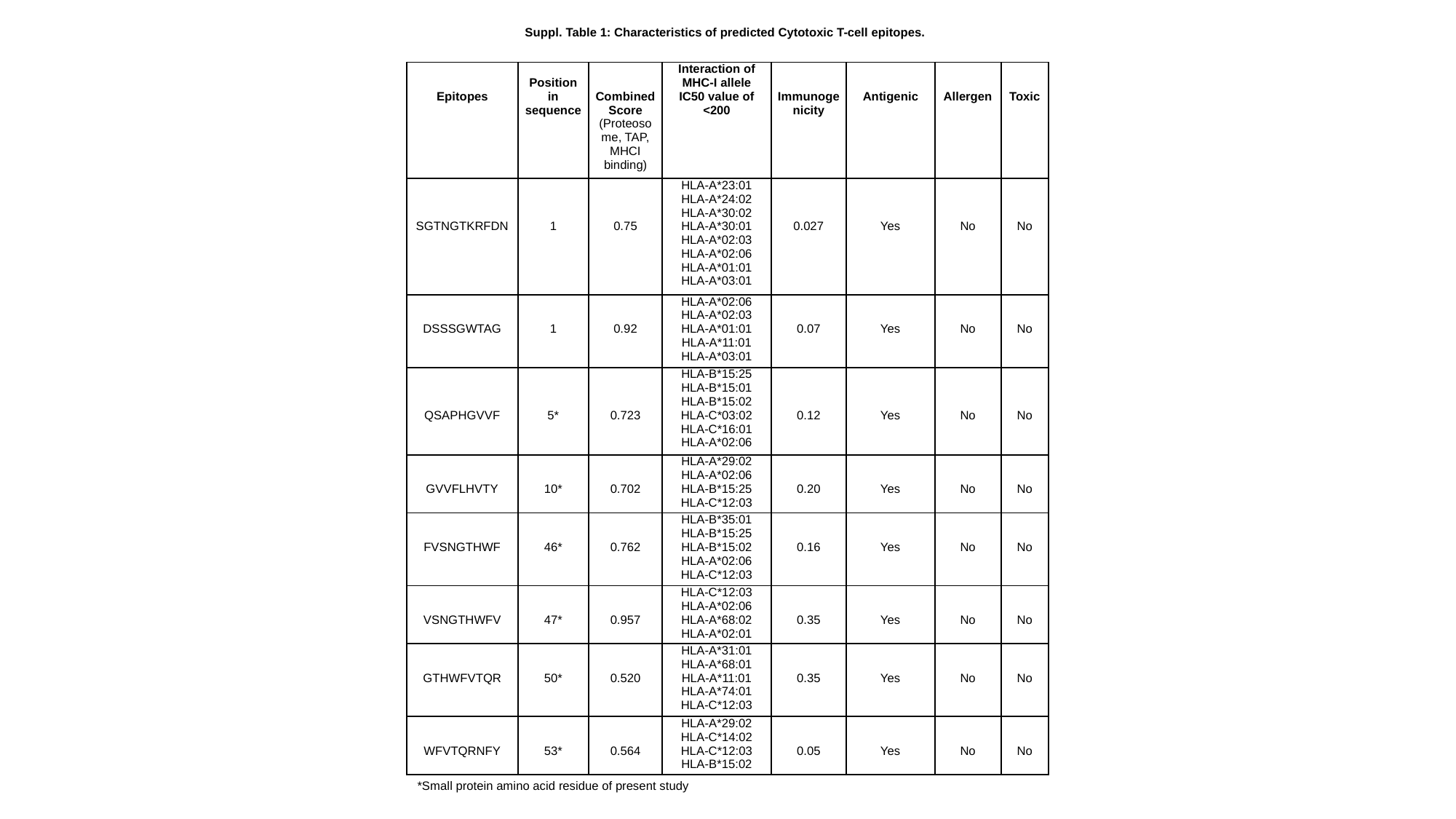

Suppl. Table 1: Characteristics of predicted Cytotoxic T-cell epitopes.
| Epitopes | Position in sequence | Combined Score (Proteosome, TAP, MHCI binding) | Interaction of MHC-I allele IC50 value of <200 | Immunogenicity | Antigenic | Allergen | Toxic |
| --- | --- | --- | --- | --- | --- | --- | --- |
| SGTNGTKRFDN | 1 | 0.75 | HLA-A\*23:01 HLA-A\*24:02 HLA-A\*30:02 HLA-A\*30:01 HLA-A\*02:03 HLA-A\*02:06 HLA-A\*01:01 HLA-A\*03:01 | 0.027 | Yes | No | No |
| DSSSGWTAG | 1 | 0.92 | HLA-A\*02:06 HLA-A\*02:03 HLA-A\*01:01 HLA-A\*11:01 HLA-A\*03:01 | 0.07 | Yes | No | No |
| QSAPHGVVF | 5\* | 0.723 | HLA-B\*15:25 HLA-B\*15:01 HLA-B\*15:02 HLA-C\*03:02 HLA-C\*16:01 HLA-A\*02:06 | 0.12 | Yes | No | No |
| GVVFLHVTY | 10\* | 0.702 | HLA-A\*29:02 HLA-A\*02:06 HLA-B\*15:25 HLA-C\*12:03 | 0.20 | Yes | No | No |
| FVSNGTHWF | 46\* | 0.762 | HLA-B\*35:01 HLA-B\*15:25 HLA-B\*15:02 HLA-A\*02:06 HLA-C\*12:03 | 0.16 | Yes | No | No |
| VSNGTHWFV | 47\* | 0.957 | HLA-C\*12:03 HLA-A\*02:06 HLA-A\*68:02 HLA-A\*02:01 | 0.35 | Yes | No | No |
| GTHWFVTQR | 50\* | 0.520 | HLA-A\*31:01 HLA-A\*68:01 HLA-A\*11:01 HLA-A\*74:01 HLA-C\*12:03 | 0.35 | Yes | No | No |
| WFVTQRNFY | 53\* | 0.564 | HLA-A\*29:02 HLA-C\*14:02 HLA-C\*12:03 HLA-B\*15:02 | 0.05 | Yes | No | No |
*Small protein amino acid residue of present study

## Slide 7
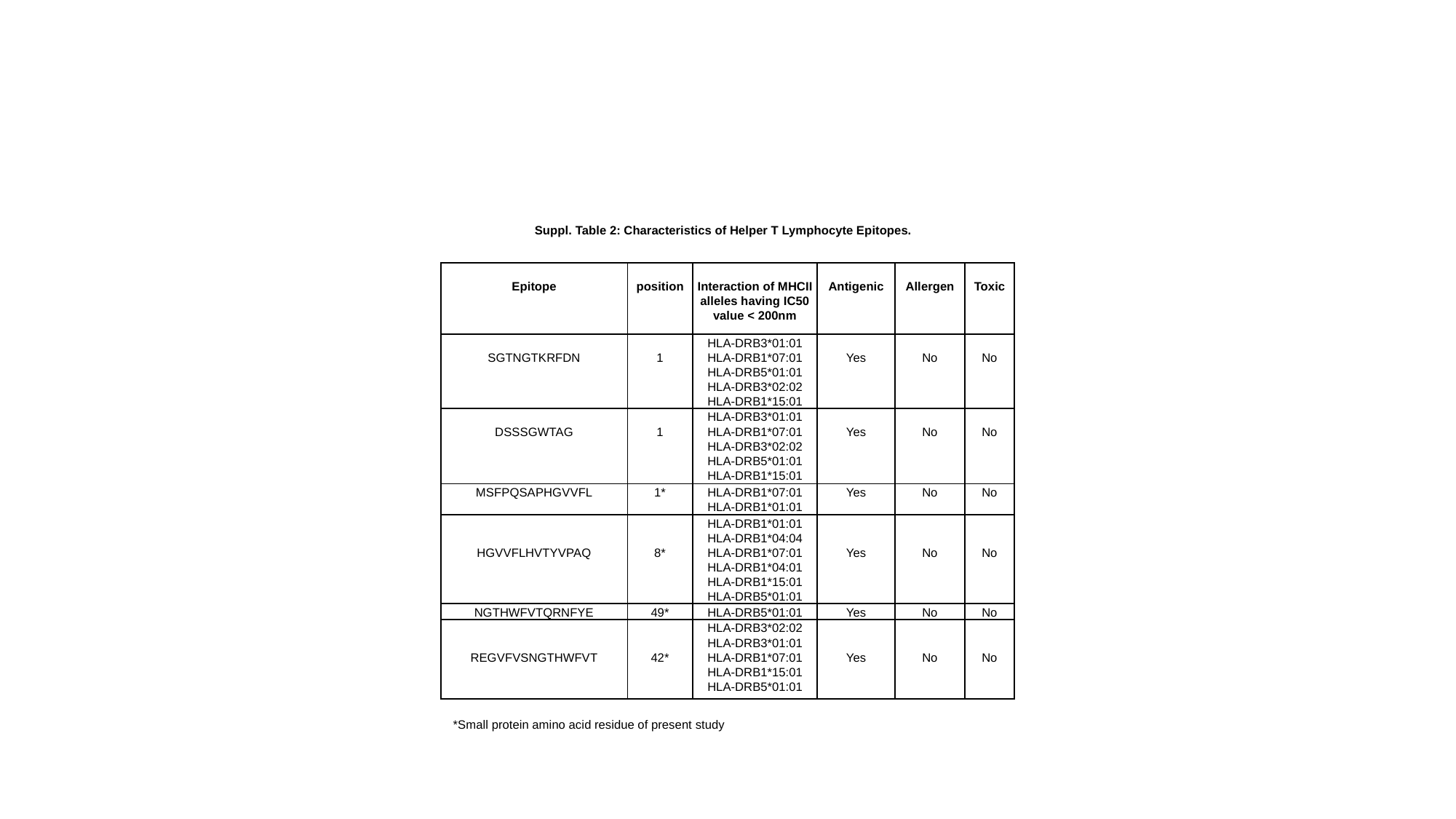

Suppl. Table 2: Characteristics of Helper T Lymphocyte Epitopes.
| Epitope | position | Interaction of MHCII alleles having IC50 value < 200nm | Antigenic | Allergen | Toxic |
| --- | --- | --- | --- | --- | --- |
| SGTNGTKRFDN | 1 | HLA-DRB3\*01:01 HLA-DRB1\*07:01 HLA-DRB5\*01:01 HLA-DRB3\*02:02 HLA-DRB1\*15:01 | Yes | No | No |
| DSSSGWTAG | 1 | HLA-DRB3\*01:01 HLA-DRB1\*07:01 HLA-DRB3\*02:02 HLA-DRB5\*01:01 HLA-DRB1\*15:01 | Yes | No | No |
| MSFPQSAPHGVVFL | 1\* | HLA-DRB1\*07:01 HLA-DRB1\*01:01 | Yes | No | No |
| HGVVFLHVTYVPAQ | 8\* | HLA-DRB1\*01:01 HLA-DRB1\*04:04 HLA-DRB1\*07:01 HLA-DRB1\*04:01 HLA-DRB1\*15:01 HLA-DRB5\*01:01 | Yes | No | No |
| NGTHWFVTQRNFYE | 49\* | HLA-DRB5\*01:01 | Yes | No | No |
| REGVFVSNGTHWFVT | 42\* | HLA-DRB3\*02:02 HLA-DRB3\*01:01 HLA-DRB1\*07:01 HLA-DRB1\*15:01 HLA-DRB5\*01:01 | Yes | No | No |
*Small protein amino acid residue of present study

## Slide 8
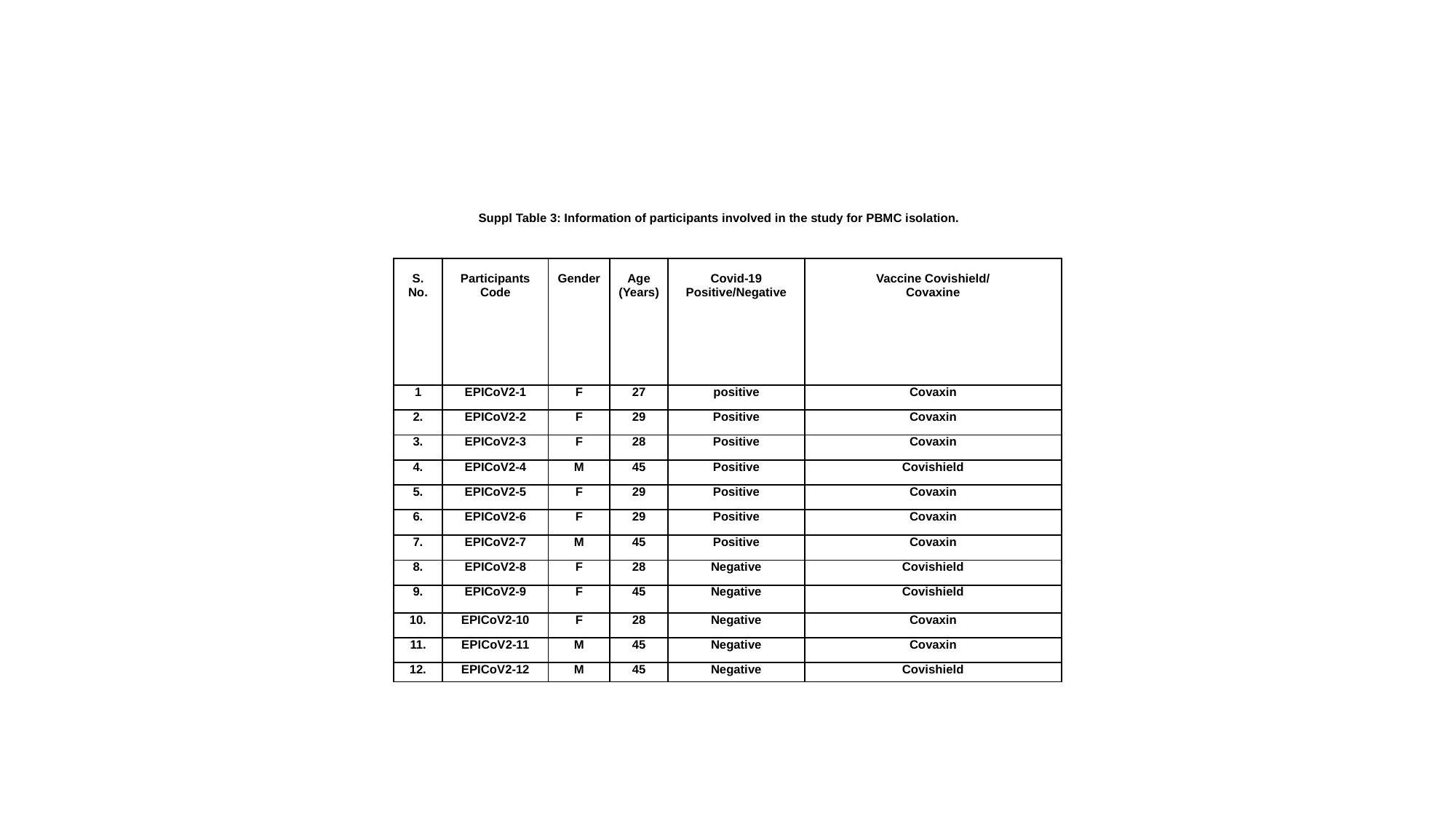

Suppl Table 3: Information of participants involved in the study for PBMC isolation.
| S. No. | Participants Code | Gender | Age (Years) | Covid-19 Positive/Negative | Vaccine Covishield/ Covaxine |
| --- | --- | --- | --- | --- | --- |
| 1 | EPICoV2-1 | F | 27 | positive | Covaxin |
| 2. | EPICoV2-2 | F | 29 | Positive | Covaxin |
| 3. | EPICoV2-3 | F | 28 | Positive | Covaxin |
| 4. | EPICoV2-4 | M | 45 | Positive | Covishield |
| 5. | EPICoV2-5 | F | 29 | Positive | Covaxin |
| 6. | EPICoV2-6 | F | 29 | Positive | Covaxin |
| 7. | EPICoV2-7 | M | 45 | Positive | Covaxin |
| 8. | EPICoV2-8 | F | 28 | Negative | Covishield |
| 9. | EPICoV2-9 | F | 45 | Negative | Covishield |
| 10. | EPICoV2-10 | F | 28 | Negative | Covaxin |
| 11. | EPICoV2-11 | M | 45 | Negative | Covaxin |
| 12. | EPICoV2-12 | M | 45 | Negative | Covishield |
